# RNF213 Variant Diversity Predisposes Distinct Populations to Dissimilar Cerebrovascular Diseases

**DOI:** 10.1155/2018/6359174

**Published:** 2018-12-20

**Authors:** Jing Lin, Wenli Sheng

**Affiliations:** Department of Neurology, First Affiliated Hospital, Sun Yat-sen University, Guanghzou, China

## Abstract

In recent years, the ring finger protein 213 gene (RNF213) has gradually attracted attention, mainly because it has been found that RNF213 c.14429 G>A is associated with moyamoya disease (MMD) in East Asian populations. Recent studies have revealed that RFN213 is not only associated with MMD but is also connected with intracranial major artery stenosis/occlusion (ICASO) and intracranial aneurysm (IA). However, only the relationship between RNF213 c.14429 G>A and ICASO has been confirmed, and whether RNF213 has other mutations related to ICASO remains unclear. RNF213 and IA are currently only confirmed to be correlated in French-Canadian Population and no correlation has been found in the Japanese population. This review summarizes the advances in the associations between RNF213 and different cerebrovascular diseases and highlights that variant diversity of RNF213 may predispose distinct populations to dissimilar cerebrovascular diseases.

## 1. Introduction

The* RNF213* gene is located on chromosome 17 and encodes a ring finger protein of 5207 amino acids. This gene has two important functional domains: a RING finger domain and an AAA+ATPase domain [1]. In recent years, RNF213 has attracted attention mainly because studies have found that RNF213 is a susceptibility gene for moyamoya disease (MMD) in East Asian populations, especially in Japanese populations [2]. Further studies have revealed that RNF213 is also connected with intracranial major artery stenosis/occlusion (ICASO) in Asian populations [3]. Zhou et al. found that different mutations of RNF213 are correlated with intracranial aneurysm (IA) in French-Canadian Population [4]. In translational medicine, partial angiogenesis was observed after knocking down* rnf213* gene in zebrafish, similar to angiogenesis in MMD [5]. However, mice with* Rnf213* gene knockout and mice with mutations corresponding to the human* RNF213* c.14429 G>A point mutation do not present phenotypes mimicking those of MMD [6, 7]. More interestingly, the overexpression of p.R4810K in human umbilical vein endothelial cells (HUVECs) inhibits angiogenesis [8], which contradicts the phenotype of moyamoya vessels partly due to angiogenesis. This review elaborates on the advances of the associations between RNF213 and different cerebrovascular diseases. RNF213 p.R4859K and RNF213 p.R4810K are amino acid variants of the same locus (rs112735431), with p.R4859K based on an in silico predicted open reading frame and p.R4810K based on the open reading frame verified by cDNA cloning. Similarly, RNF213 c.14429 G>A and RNF213 c.14576 G>A correspond to the same single nucleotide variation. RNF213 p.R4810K and RNF213 c.14429 G>A are used consistently in this review.

## 2. RNF213 and MMD

### 2.1. Genetic Factors Involved in MMD, Especially RNF213

MMD is a rare cerebrovascular disease and is one of the main causes of stroke in children. It is primarily characterized by the progressive stenosis of the internal carotid artery and an abnormal vascular network at the base of the brain [9]. Thickening of the tunica intima and thinning of the media is the main pathological feature of MMD [10], but the pathogenesis of MMD remains unclear. Some MMD patients present autosomal dominant inheritance, and MMD is more common in Asians than in Europeans, suggesting that genetic factors may be involved in the pathogenesis of MMD. It has been found that multiple loci are associated with MMD: 3p24-p26, 6q25, 8q23, and 17q25 [11–14]. Kamada et al. found that* RNF213* at 17q25 is a new susceptibility gene in East Asian MMD patients. The polymorphism c.14429 G>A of this gene is present in 95% of familial MMD and in 79% of sporadic MMD patients [2]. This suggests that genetic factors are involved in the pathogenesis of MMD, especially RNF213.

### 2.2. Clinical Studies

#### 2.2.1. RNF213 p.R4810K and Distinct Populations

There is a significant racial difference in the correlation between RNF213 p.R4810K and MMD. This mutation is found in 90.1% of Japanese MMD patients, in 78.9% of Korean MMD patients, and in 23.1% of Chinese MMD patients. Normal populations also have this variation, which is found in 2.5% of Japanese, 2.7% of Korean, and 0.9% of Chinese populations [5]. Compared with Japanese and Korean patients, the rate of this mutation in Chinese Han MMD patients is lower [15]. The incidence of MMD in Europeans is about 1/10 of that found in Japanese [16], and RNF213 p.R4810K was not identified in Europeans [5], which may be one of the reasons for the low incidence of MMD in Europeans. In a study of RNF213 p.R4810K and MMD patients with different descent living in the similar environment, p.R4810K was found in 56% of Asian descent MMD patients and not found in non-Asian descent MMD patients [17]. Our previous meta-study found that p.R4810K is associated with MMD, and compared with China, the association was more prominent in Japan and Korea. Additionally, p.A4399T was not associated with Asian MMD patients, and p.A5021V was only related to Chinese Han MMD patients [18]. This suggests that RNF213 p.R4810K involved in the pathogenesis of MMD is ethnically diverse.

#### 2.2.2. RNF213 R4810K Homozygote/Heterozygote and MMD

It has been found that the dose effect of RNF213 is correlated to the presence of MMD. Miyatake et al. found that the homozygous mutation of RNF213 p.R4810K was only present in patients with MMD and was not found in normal populations, and homozygous mutants showed earlier disease onset and more severe conditions than did heterozygous mutants [19]. However, later studies have confirmed that RNF213 p.R4810K homozygous mutations also exist in normal people [20], and twins with the same genetic background can present different phenotypes [5]. This suggests that the MMD phenotype cannot be explained solely by gene dose effects.

### 2.3. Basic Research

#### 2.3.1. HUVECs E2488Q Mutants Corresponding to p.R4810K Inhibit Angiogenesis

RNF213 has two important functional domains: the RING finger domain and the AAA+ATPase domain. The AAA+ATPase domain has two AAA+ modules. The first module is essential for assembling RNF213 oligomers, whereas the second module contributes to disassemble RNF213 oligomers. The oligomeric state is initiated by ATP binding to the Walker A motif in the first AAA+ module and the Walker B motif in the second AAA+ module can hydrolyze ATP to disassemble oligomers [1]. Kobayashi et al. found that the point mutation in the Walker B motif of the first AAA+ module of RNF213 (E2488Q) similar to the RNF213 p.R4810K mutation decreases ATPase activity and stabilizes oligomers, thereby inhibiting angiogenesis. However, the deletion mutation of the first AAA+ module of RNF213 (RNF213 delAAA) does not inhibit angiogenesis, similar to the wild type. The Walker B motif point mutation (E2488Q) of the AAA+ module can maintain oligomers, and the AAA+ module deletion mutation (RNF213 delAAA) cannot maintain the oligomeric state [8]. This suggests that the RNF213 p.R4810K mutant inhibits ATP hydrolysis to maintain the oligomeric state, thereby inhibiting angiogenesis.

#### 2.3.2. Mouse* Rnf213* Knockout or p.R4828K Mutant Does Not Completely Mimic the Phenotype of MMD

Clinical studies suggest that RNF213 is associated with MMD. Since there is no suitable animal model for MMD, many studies have attempted to establish MMD models based on* RNF213* gene knockout or point mutations. Sonobe et al. used the cre-lox system to knockout exon 32 of mouse* Rnf213*, but the resulting mice did not show intracranial artery stenosis and smog-like vascular phenotypes. They also did not show phenotypes mimicking those related to MMD even when superimposed with hypoxic environmental factors (by occluding the carotid artery); however, the common carotid artery showed transient intimal and medial thinning [6]. Kanoke et al. used the cre-lox system to generate the mouse Rnf213 p.R4828K point mutation (corresponding to the human p.R4810K point mutation), which does not show phenotypes mimicking those related to MMD even when superimposed with hypoxia [7]. In addition, Kanoke et al. gave* Rnf213* exon 32 knockout mice a strong dose of immunoadjuvant, which did not mimic the phenotype of MMD [21]. Ito et al. found that the* Rnf213* exon 32 knockout mice showed significantly enhanced angiogenesis after long-term ischemia [22]. Studies on a variety of mice specifically overexpressing RNF213 p.R4757K (corresponding to the human p.R4810K locus) revealed that a hypoxic environment promoted angiogenesis, but angiogenesis in mice in which endothelial cells overexpressed RNF213 p.R4757K was significantly inhibited [8]. This suggests that* Rnf213* gene knockout promotes angiogenesis, but p.R4810K point mutation inhibits angiogenesis. After transient occlusion of the middle cerebral artery in rats, Sato-Maeda et al. found a significant increase in RNF213 expression in the ischemic penumbra, which showed association with apoptotic neurons [23]. This group later briefly clamped the rat common carotid artery to cause general cerebral ischemia and found that neuronal apoptosis in hippocampal CA1 region was associated with elevated* Rnf213* mRNA [24], suggesting that RNF213 mediates apoptosis of hypoxic-ischemic neurons.

#### 2.3.3. Zebrafish* rnf213* Knockdown or Knockout Partly Mimics the Phenotype of MMD

The entire cerebral blood vessels of zebrafish can be clearly observed after inhibiting the formation of melanin. Therefore, Liu et al. used zebrafish as an animal model and found that knockdown of* rnf213* expression by morpholino could promote angiogenesis and partially mimic smog-like blood vessels [5]. However, the phenotypes produced through morpholino-mediated knockdown of* rnf213* expression in zebrafish may have been affected by off-target effects, and recent studies have found that morpholinos have a high probability of off-target effects [25, 26]. Therefore, we knocked out zebrafish* rnf213* via a transcription activator-like effector nuclease and found significant angiogenesis of the intersegmental blood vessels and cerebral blood vessels and small blood vessel stenosis in the F0 generation [27]. By similarly knocking out RNF213, the zebrafish model can partially mimic the MMD phenotype, but mouse models failed to mimic the MMD phenotype, even if the mice were superimposed with hypoxia or immunoadjuvant, suggesting that RNF213 may play different roles in MMD in different species.

## 3. RNF213 and ICASO

Recent studies found that RNF213 p.R4810K is not only related to MMD but is also related to non-MMD ICASO. Miyawaki et al. found that the RNF213 p.R4810K point mutation was present in 9 of 41 ICASO patients [3]. To confirm this finding, Miyawaki et al. conducted an expanded sample size study and found the RNF213 p.R4810K point mutation in 20/84 ICASO patients [28]. Bang et al. found that 176/352 ICASO patients had RNF213 p.R4810K point mutations [29]. Shinya et al. found that RNF213 p.R4810K is associated with anterior circulation ICASO and is not associated with posterior circulation ICASO or extracranial carotid atherosclerosis [30]. Yeung et al. found a significant association between RNF213 p.R4810K and the ICASO phenotype in CADASIL (cerebral autosomal dominant arteriopathy with subcortical infarcts and leukoencephalopathy) patients [31]. Liao et al. conducted a meta-analysis, summarizing 11 studies of p.R4810K and ICASO (including 1778 ICASO patients and 3140 normal controls) and found that p.R4810K was significantly associated with ICASO (OR 13.89, 95% CI 8.01–24.09, and p < 0.0001) [32]. The above studies suggest that RNF213 p.R4810K is correlated with ICASO. However, studies on the correlation between RNF213 and ICASO have only reported one locus of p.R4810K, and correlations between RNF213 other variant and ICASO have not been found up to now.

## 4. RNF213 and IA

It has been found that French-Canadian population has a higher incidence of IA, and IA patients are often found in families, especially large families. Zhou et al. found that two RNF213 point mutations (p.R2438C and p.A2826T) were associated with intracranial aneurysms in French-Canadian Population, both of which are located in the AAA+ATPase domain, and ATPase activity was increased in IA patients. It is thus speculated that RNF213 p.R2438C and RNF213 p.A2826T cause an increase in ATPase activity to promote angiogenesis and participate in the formation of IA [4]. However, Miyawaki et al. found that RNF213 p.R4810K was not significantly associated with IA patients of Japanese descent, but no other site mutations were examined [3, 28]. This suggests that different mutation sites may be involved in the pathogenesis of IA.

## 5. RNF213 Variant Diversity Is Associated with Different Phenotypes 

**Figure 1:**
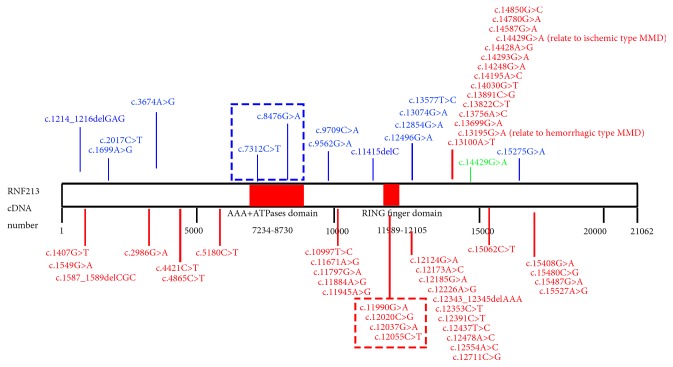
**RNF213 Nucleotide Variants Found In MMD, IA, and ICASO. **Variants associated with MMD are marked in red; variants associated with IA are marked in blue; variants associated with ICASO are marked in green; red box represent variants in RING finger domain related to MMD; blue box represent variants in AAA+ATPases domain related to IA. Two special variants (c.13195G>A and c.14429G>A) are associated with different type MMD, respectively.

Various* RNF213* genetic mutations related to cerebrovascular disease have been reported (Table 1). These mutations are predominantly missense mutations, and most of the point mutation sites are located at the C-terminus (Figure 1). Mutations in different sites of RNF213 may have different effects on blood vessels. The RNF213 p.R4810K mutation may be the main cause of MMD intracranial artery stenosis [3, 33]. It has been found that the RNF213 p.A4399T mutation is more related to the MMD bleeding phenotype, while RNF213 p.R4810K is more related to the MMD ischemic phenotype [15]. Two reported mutation sites in the AAA+ATPase domain (c.7312 C>T and c.8476 G>A) are related to IA [4], and four reported mutation sites in the RING finger domain (c. 11990 G>A, c.12020 C>G, c.12037 G>A, and c.12055 C>T) are all related to MMD [15, 17, 34]. This suggests that the AAA+ATPase domain may be more related to IA, and the RING finger domain may be more relevant to MMD. In addition to point mutations, it has been found that four frameshift mutations in* RNF213* are also associated with cerebrovascular disease, with c.1214_1216delGAG and c.11415delC associated with aneurysms [4] and c.1587_1589delCGC and c.12343_12345delAAA associated with MMD [17]. Frameshift mutations often cause loss function of the entire protein, but the three reported frameshift mutations of* RNF213* (c.1214_1216delGAG, c.1587_1589delCGC, c.12343_12345delAAA) all cause the missing of 3bp, which may have little effect on the whole protein function. The c.11415delC frameshift mutation only deletes 1bp and is located in front of the RING finger domain, which may result in loss of the RING finger domain function.

## 6. Conclusion

There are significant racial differences in the correlations of* RNF213* with MMD and IA. The correlation between RNF213 p.R4810K and MMD is reported in Asian populations but not identified in Europeans and non-Asian descent Americans. Similarly, the correlation between RNF213 and IA was confirmed only in French-Canadian Population, and no correlation was found in the Japanese population. Different site mutations in RNF213 may be involved in different cerebrovascular diseases. Current ICASO studies report that only RNF213 p.R4810K is associated with ICASO, and other mutation sites have not been reported. In addition to RNF213 p.R4810K, many RNF213 sites have been reported to be associated with MMD, and RNF213 p.R4810K is more related to MMD patients in East Asia. In terms of RNF213 sites associated with IA, only two mutations (p.R2438C and p.A2826T) have been reported. Different mutation sites of RNF213 are associated with different cerebrovascular diseases, possibly because different mutations affect different functional domains of RNF213. Therefore, RNF213 variant diversity predisposes distinct populations to dissimilar cerebrovascular diseases. Unfortunately, mice with* Rnf213* knockout or point mutations similar to those of humans do not fully mimic the MMD phenotype. Environmental aspects may also be important factors in MMD pathogenesis. More studies are required to confirm the correlation of RNF213 with various cerebrovascular diseases, thus providing a new target for the prevention and treatment of cerebrovascular diseases.

## Figures and Tables

**Table 1:** RNF213 variants diversity in cerebrovascular diseases (Italic represents variant associated with hemorrhagic type MMD; bold represents variant related to ischemic type MMD).

**Diseases**	**Nucleotide **	**Amino acid**	**Reference**
**MMD**	c.1407G>T	p.Q469H	Schilter (2017) Am J Med Genet A 173, 2557
	c.1549G>A	p.G517R	Shoemaker (2015) G3 (Bethesda) 6, 41
	c.1587_1589delCGC		Cecchi (2014) Stroke 45, 3200
	c.2986G>A	p.E996K	Akagawa (2018) Hum Genome Var 5, 17060
	c.4421C>T	p.S1474F	Shoemaker (2015) G3 (Bethesda) 6, 41
	c.4865C>T	p.A1622V	Lee (2015) J Neurol Sci 353, 161
	c.5180C>T	p.T1727M	Zhang (2017) J Neurosurg 126, 1106
	c.10997T>C	p.M3666T	Shoemaker (2015) G3 (Bethesda) 6, 41
	c.11671A>G	p.M3891V	Kamada (2011) J Hum Genet 56, 34
	c.11797G>A	p.V3933M	Lee (2015) J Neurol Sci 353, 161
	c.11884A>G	p.N3962D	Liu (2011) PLoS One 6, e22542
	c.11945A>G	p.K3982R	Shoemaker (2015) G3 (Bethesda) 6, 41
	c.11990G>A	p.C3997Y	Cecchi (2014) Stroke 45, 3200
	c.12020C>G	p.P4007R	Wu (2012) PLoS One 7, e48179
	c.12037G>A	p.D4013N	Cecchi (2014) Stroke 45: 3200
	c.12055C>T	p.R4019C	Kobayashi (2016) PLoS One 11: e0164759
	c.12124G>A	p.E4042K	Kobayashi (2016) PLoS One 11, e0164759
	c.12173A>C	p.H4058P	Akagawa (2018) Hum Genome Var 5, 17060
	c.12185G>A	p.R4062Q	Moteki (2015) J Am Heart Assoc 4: e001862
	c.12226A>G	p.I4076V	Cecchi (2014) Stroke 45, 3200
	c.12343_12345delAAA		Cecchi (2014) Stroke 45, 3200
	c.12353C>T	p.S4118F	Harel (2015) Am J Med Genet A 167, 2742
	c.12391C>T	p.R4131C	Lee (2015) J Neurol Sci 353, 161
	c.12437T>C	p.V4146A	Kobayashi (2016) PLoS One 11, e0164759
	c.12478A>C	p.K4160Q	Zhang (2017) J Neurosurg 126, 1106
	c.12554A>C	p.K4185T	Smith (2014) Int J Stroke 9, E26
	c.12711C>G	p.D4237E	Cecchi (2014) Stroke 45, 3200
	c.13100A>T	p.Q4367L	Wu (2012) PLoS One 7, e48179
	*c.13195G>A*	*p.A4399T*	*Wu (2012) PLoS One 7, e48179*
	c.13699G>A	p.V4567M	Kamada (2011) J Hum Genet 56, 34
	c.13756A>C	p.T4586P	Wu (2012) PLoS One 7, e48179
	c.13822C>T	p.P4608S	Liu (2011) PLoS One 6, e22542
	c.13891C>G	p.L4631V	Wu (2012) PLoS One 7, e48179
	c.14030G>T	p.W4677L	Schilter (2017) Am J Med Genet A 173, 2557
	c.14195A>C	p.K4732T	Cecchi (2014) Stroke 45, 3200
	c.14248G>A	p.E4750K	Moteki (2015) J Am Heart Assoc 4, e001862
	c.14293G>A	p.V4765M	Kamada (2011) J Hum Genet 56, 34
	c.14428A>G	p.R4810G	Shoemaker (2015) G3 (Bethesda) 6, 41
	**c.14429G>A**	**p.R4810K**	**Kamada (2011) J Hum Genet 56, 34**
	c.14587G>A	p.D4863N	Liu (2011) PLoS One 6, e22542
	c.14780G>A	p.R4927Q	Moteki (2015) J Am Heart Assoc 4, e001862
	c.14850G>C	p.E4950D	Liao (2017) Environ Health Prev Med 22: 75
	c.15062C>T	p.A5021V	Liu (2011) PLoS One 6, e22542
	c.15408G>A	p.M5136I	Wu (2012) PLoS One 7, e48179
	c.15480C>G	p.D5160E	Liu (2011) PLoS One 6, e22542
	c.15487G>A	p.V5163I	Cecchi (2014) Stroke 45, 3200
	c.15527A>G	p.E5176G	Liu (2011) PLoS One 6, e22542

**IA**	c.1214_1216delGAG		Zhou (2016) Am J Hum Genet 99, 1072
	c.1699A>G	p.M567V	Zhou (2016) Am J Hum Genet 99, 1072
	c.2017C>T	p.R673W	Zhou (2016) Am J Hum Genet 99, 1072
	c.3674A>G	p.D1225G	Zhou (2016) Am J Hum Genet 99, 1072
	c.7312C>T	p.R2438C	Zhou (2016) Am J Hum Genet 99, 1072
	c.8476G>A	p.A2826T	Zhou (2016) Am J Hum Genet 99, 1072
	c.9562G>A	p.V3188M	Zhou (2016) Am J Hum Genet 99, 1072
	c.9709C>A	p.Q3237K	Zhou (2016) Am J Hum Genet 99, 1072
	c.11415delC		Zhou (2016) Am J Hum Genet 99, 1072
	c.12496G>A	p.D4166N	Zhou (2016) Am J Hum Genet 99, 1072
	c.12854G>A	p.S4285N	Zhou (2016) Am J Hum Genet 99, 1072
	c.13074G>A	p.K4358=	Zhou (2016) Am J Hum Genet 99, 1072
	c.13577T>C	p.I4526T	Zhou (2016) Am J Hum Genet 99, 1072
	c.15275G>A	p.R5092Q	Zhou (2016) Am J Hum Genet 99, 1072

**ICASO**	c.14429G>A	p.R4810K	Miyawaki (2012) Stroke 43, 3371
			Miyawaki (2013) Stroke 44, 2894
